# Pediatric adenoidectomy is safe surgery with a low complication rate: a population-based study

**DOI:** 10.1038/s41598-025-13803-9

**Published:** 2025-07-31

**Authors:** Hannah Losgar, Daniel Boeger, Jens Buentzel, Kerstin Hoffmann, Jiri Podzimek, Holger Kaftan, Andreas Mueller, Sylvia Tresselt, Katharina Geißler, Orlando Guntinas-Lichius

**Affiliations:** 1 Department of Otorhinolaryngology, Jena University Hospital, Jena, Germany; 2 Department of Otorhinolaryngology, Zentralklinikum, Suhl, Germany; 3 Department of Otorhinolaryngology, Südharz-Krankenhaus gGmbH, Nordhausen, Germany; 4 Department of Otorhinolaryngology, Sophien/Hufeland-Klinikum, Weimar, Germany; 5 Department of Otorhinolaryngology, Klinikum Bad Salzungen, Bad Salzungen, Germany; 6 Department of Otorhinolaryngology, Helios-Klinikum, Erfurt, Germany; 7 Department of Otorhinolaryngology, SRH Wald-Klinikum, Gera, Germany; 8 Department of Otorhinolaryngology, Ilm-Kreis-Kliniken, Arnstadt, Germany; 9 Department of Otorhinolaryngology, Jena University Department, Am Klinikum 1, D-07747 Jena, Germany

**Keywords:** Adenoidectomy, Tonsillectomy, Tonsillotomy, Complications, Clavien-Dindo classification, Incidence rate, Surgical rate, Epidemiology, Medical research, Paediatric research, Signs and symptoms, Comorbidities

## Abstract

Population-based data on incidence of complications after pediatric adenoidectomy are sparse. Therefore, a retrospective population-based study of all 2105 pediatric adenoidectomies (59.9% male, median age: 4 years) in the year 2019 in all otolaryngology departments in one federal state, Thuringia, in Germany, was performed. Patients’ and treatment characteristics, and complications were analyzed. The highest surgery rate was seen at the age of 3 years (2747.4 per 100,000 children). Adenoidectomy was combined with tonsillotomy or tonsillectomy in 29.2% and 1.5% of the cases. Postoperative bleeding needing re-surgery occurred in 1.1% of all cases. The revision surgery for bleeding rate after solitary adenoidectomy was 0.7%. A wound infection was seen in 1.0%. Complications classified according to the Clavien-Dindo classification (CDC) occurred in 2.6% of cases. The overall complication rate was 20.3/100,000 population. Additional tonsillectomy was independently associated to bleeding > 24 h after surgery (Odds ratio [OR] = 52.141; confidence interval [CI] = 7.772-349.818; *p* < 0.0001). There was no independent associative factor to enhanced risk of wound infection. CDC complications occurred more frequently in comorbid patients (OR = 4.175; CI = 1.222–14.271; *p* = 0.023), underweight children (OR = 2.430; CI = 1.198–6.571; *p* = 0.040), when additional tonsillectomy was performed (OR = 11.177; CI = 2.098–59.548; *p* < 0.0001), and when perioperative antibiotics were applied (OR = 13.251; CI = 5.695–30.834; *p* < 0.001). Adenoidectomy is very safe surgery. Main risk factor for bleeding complications is additional tonsillectomy, not adenoidectomy itself.

**Supplementary Information:**

The online version contains supplementary material available at 10.1038/s41598-025-13803-9.

## Introduction

Adenoidectomy is one of the most commonly performed pediatric surgeries. The two most common indications are adenoidectomy alone or as part of a combined procedure with tonsil surgery for the management of pediatric sleep-related disorders or in association with ventilating tube placement in the management of pediatric otitis media with effusion^1,2^. Although adenoidectomy is that common, most knowledge on outcome and complications comes from hospital-based data^3,4^. In contrast, population-based data on adenoidectomy, and especially of perioperative risk factors are sparse. There are national register-based data from Sweden analyzing 40,829 adenoidectomies during 2004 to 2013 ^5^. Surgery was mainly performed on the indication adenoid hypertrophy, mainly between 2 and 5 years of age and that the incidence in surgical rates was higher for boys than girls. The Swedish study was recently updated with complication data^5^. In this second analysis, cases with additional tonsil surgery were excluded. For 51,746 solitary adenoidectomies during 2007 to 2017, Only 0.1% of the surgeries resulted in an outpatient contact due to postoperative hemorrhage and only 0.1% of the adenoidectomies resulted in a readmission due to hemorrhage. This is, to our knowledge, so far the only large population-based study on complications after adenoidectomy. A Taiwanese population-level analysis of 10,396 children was exclusively focused on the re-adenoidectomy rate^6^. In 2013 we also published population-based data on the incidence rate of re-adenoidectomy in 2009 ^8^. Thuringia is a territorial state in Germany with approximately 2.2 million inhabitants. There are only eight hospitals with departments of otorhinolaryngology in Thuringia. The departments of otorhinolaryngology have built a network primarily to improve health services research in the field of otorhinolaryngology. Use of this network provides an ideal platform for a population-based analysis of otorhinolaryngology patients^7–9^.

We hypothesized that pediatric adenoidectomy is a safe procedure. Therefore, the present study wanted to up-date the data on epidemiology and incidence rates of pediatric adenoidectomy, but also to add data on complications after adenoidectomy and associated factors from a population-based perspective.

## Methods

### Study design and setting

The institutional ethics committee of the Jena University Hospital, Jena, Germany, approved the study protocol for a retrospective data collection. Only anonymized data were analyzed. Therefore, the ethics committee of the Jena University Hospital, Jena, Germany, waived the need for written informed consent. All methods were performed in accordance with the relevant guidelines and regulations. A standardized retrospective analysis was performed in all eight Thuringian hospitals with a department of otolaryngology (in alphabetic order: Arnstadt, Bad Salzungen, Erfurt, Gera, Jena, Nordhausen, Suhl, Weimar).

### Patients

All children (0–17 years of age) were selected who received an adenoidectomy (coded by 5-285.0, 5-285.1, 5-282, 5-2820, 5-282.1 due to operation and procedure classification system [OPS]) alone or in combination with other procedures and who were treated in 2019. 2105 children were identified and included into the study. A retrospective search of the patients’ charts was performed. The following variables were obtained: age, sex, body mass index (BMI), comorbidity classified by Charlson comorbidity index (CCI^10^;, and data of all surgical procedures. Due to the Kromeyer-Hauschild BMI reference system for German children, the 90th, 97th, and 99.5th percentile of the BMI was used to define overweight, obesity, and extreme obesity separately for male and female patients^11,12^. The classification of the constriction of the oropharynx by the tonsils based on the Brodsky grading scale^13^. Specific complications were recorded and also all complications defined by the Clavien-Dindo classification (CDC)^14^. Primary postoperative bleeding was defined as bleeding within 24 h. Secondary bleeding was defined as bleeding later than 24 h after surgery.

### Epidemiology

The epidemiological calculations for the incidence of adenoidectomy (surgical rate) per 100,000 population per age and gender were based on the annual mean number of habitants in Thuringia in 2019. Population numbers of the online database of the Thuringian State Office for Statistics (www.tls.thueringen.de) were used.

### Statistical analysis

Participants’ characteristics and outcome variables were analyzed with IBM SPSS statistics software (Version 28.0.0.0) for medical statistics. Data are presented as mean ± standard deviation (SD) if not otherwise indicated. The chi-square test was used to compare nominal data of two independent subgroups. Fisher’s exact test was used to compare ordinal data of > 2 independent subgroups. The Mann-Whitney U-test was used to compare scaled data of two independent subgroups. Binary logistic regression models were generated to determine odds ratios (OR) and 95% confidence intervals (CI) for the analysis of associations between patients’ and treatment characteristics and the occurrence of complications and risk of re-adenoidectomy. Patients’ characteristics for regression analysis were derived from those variables that were significant in preliminary univariate analyses (*p* < 0.05). Multivariate regression were calculated for the occurrence of any complication (model 1), bleeding after 24 h (model 2), wound infection (model 3), CDC complications (model 4), and later re-adenoidectomy (model 5). The results are presented in odds ratio (OR) and confidence intervals (CI). Because only one parameter showed a significant association to bleeding within the first 24 h, a multivariate analysis was unnecessary here. Patients with missing values were omitted from univariate or multivariate subanalysis on these parameters. This was only important for the parameter BMI because this was missing for 30.7% of the patients. This missing appeared to occur at random. There was no difference in age, gender, comorbidity, and surgery type between patients with available BMI and the patients with missing BMI (all *p* > 0.05). In general, nominal p values of two-tailed tests are reported. The significance level was set to *p* < 0.05.

## Results

### Study participants and treatment characteristics

Two thousand and one hundred and five (2105) adenoidectomies were performed in 2019 in Thuringia. Patients’ characteristics are summarized in Table 1. Eight hundred and forty-eight (844) female and 1261 male participants (male to female ratio: 1.5:1) were analyzed. The median age was 4 years. The median BMI was 15.5. 8.9% of the children were overweight, and 10.5% obese. 4.7% had a comorbidity due the Charlson Comorbidity Index. The majority had a palatine tonsil size Brodsky grade II/III (57.6%). Table 2 shows the treatment details. Adenoidectomy alone was the most frequent surgery (69.3%), followed by adenoidectomy plus tonsillotomy (29.2%), and rarely adenoidectomy plus tonsillectomy (1.5%). Most adenoidectomies were performed as conventional adenoidectomy with curette (99.5%). Only two cases (0.1%) were performed with coblation adenoidectomy and eight cases (0.4%) with suction diathermy adenoidectomy.

**Table 1:** Patients’ characteristics.

Parameter	*n*	%
All	2105	100
Gender		
Male	1261	59.9
Female	844	40.1
Charlson Comorbidity Index (CCI)*		
0 (no comorbidity)	2008	95.4
1	73	3.5
2	20	1.0
3	2	0.1
4	2	0.1
Age and gender specific percentiles of the BMI		
3.0 (underweight)	127	6.0
10.0	152	7.2
25.0	235	11.2
50.0	272	12.9
75.0	265	12.6
90.0 (overweight)	187	8.9
97.0 (obesity)	95	4.5
99.5 (extreme obesity)	126	6.0
Unknown	646	30.7
Brodsky grade		
No obstruction, grade 0	497	23.6
≤ 25% obstruction, grade I	73	3.5
26–50% obstruction, grade II	587	27.9
51–75% obstruction, grade III	626	29.7
> 75% obstruction, grade IV	173	8.2
Unknown	149	7.1
Indication for surgery (ICD-Codes)**		
All ICD codes	3886	100
Adenoid hyperplasia (J35.2)	1727	43.1
Otitis media with effusion (H65)	1313	32.7
Adenoid and tonsil hyperplasia J35.3)	380	9.5
Tonsil hyperplasia (J35.1)	255	6.4
Eustachian tube dysfunction (H69.8)	128	3.2
Obstructive sleep apnea (G47.31)	48	1.2
Chronic tonsillitis (J35.0)	35	0.9
	**Mean ± SD**	**Median**,** range**
Age, years	4.2 ± 2.6	4, 0.1–17
Size (cm), *n* = 1463	108 ± 19	105, 50–190
Weight (kg), *n* = 1795	20 ± 12	17, 7-140
BMI, *n* = 1459	16.3 ± 3.9	15.5, 7.0-48.1

*The CCI includes 19 conditions. The maximal score is 37; **sum higher than number of patients; SD = standard deviation; BMI = Body-Mass-Index.

**Table 2:** Treatment characteristics.

Parameter	*n*	%
All	2105	100
Setting		
Inpatient	1968	93.5
Outpatient	137	6.5
Surgery		
Adenoidectomy, solitary	1459	69.3
Adenoidectomy + tonsillotomy	614	29.2
Adenoidectomy + tonsillectomy	32	1.5
Additional surgeries		
Myringotomy and ventilation tubes	461	21.9
Myringotomy	392	18.6
Perioperative antibiotics		
Yes	97	4.6
No	2008	95.4
	**Mean ± SD**	**Median**,** range**
Outpatient/inpatient duration, days	1.7 ± 1.3	1, 0–14
Surgery time, min (*n* = 1801)	34 ± 19	30, 14–212

SD = standard deviation.

### Complications of adenoidectomy and later re-adenoidectomy rate

The complications are summarized in Table 3. Twenty primary bleeding events (1.0% of all surgeries) and 25 secondary bleedings (1.2%) occurred. One child had a primary and secondary bleeding, i.e. a postoperative bleeding occurred in 44 children (bleeding rate: 2.1%). A surgical revision was needed for 23 out the 44 children, i.e. in about the half of the bleeding cases (revision surgery for bleeding rate: 1%). Revision surgery was needed for 14 primary bleedings and for 10 secondary bleedings. The one child with combined primary and secondary bleeding needed surgery for both events. The revision surgery for bleeding rates after solitary adenoidectomy, adenoidectomy plus tonsillotomy, and adenoidectomy plus tonsillectomy were 0.7%, 1.6%, and 9.4%. The source of bleeding was the tonsil surgery in 12 cases, and the adenoid surgery in 11 cases. In case of combined adenoid and tonsil surgery, the bleeding needing revision surgery was related to the prior tonsil surgery in 12 out of 13 events. A wound infection was seen in 1% of the patients. Other severe adverse events did not occur. When using the Clavien-Dindo classification, this results in grade 0, I, II, and IIIb in 97.4%, 1.2%, 0.6%, and 0.7% of the cases, respectively.

**Table 3:** Complications and complication management.

Parameter	*n*	%
All	2105	100
Bleeding within 24 h		
Yes	20	1.0
After adenoidectomy, solitary	14	
After adenoidectomy + tonsillotomy	9	
After adenoidectomy + tonsillectomy	0	
No	2085	99.0
Bleeding after 24 h		
Yes	25	1.2
After adenoidectomy, solitary	7	
After adenoidectomy + tonsillotomy	12	
After adenoidectomy + tonsillectomy	6	
No	2080	98.8
Bleeding, any*		
Yes	44	2.1
After adenoidectomy, solitary	21	1.0
After adenoidectomy + tonsillotomy	17	0.8
After adenoidectomy + tonsillectomy	6	0.3
No	2061	97.7
Wound infection		
Yes	22	1.0
No	2083	99.0
Tooth damage		
Yes	2	0.1
No	2103	99.9
Specific complications, cumulative		
Yes	66	3.1
No	2039	96.9
Nerve damage, Grisel syndrome, hypogeusia, death		
Yes	0	0
No	2195	100
Clavien-Dindo-classification		
Grade 0	2051	97.4
Grade I	26	1.2
Grade II	13	0.6
Grade IIIb	15	0.7
Bleeding therapy**		
No bleeding	2038	96.8
Wait-and-see	24	1.1
Re-surgery for hemostasis	23	1.1
Bleeding source: tonsil surgery	12	0.6
Bleeding source: adenoid surgery	11	0.5
After adenoidectomy	10	0.5
After adenoidectomy + tonsillotomy	10	0.5
After adenoidectomy + tonsillectomy	3	0.1
Fluid substitution	18	0.9
Iron substitution	1	0.05
Transfusion	1	0.05
Re-admission because of a complication		
Yes	27	1.3
No	2078	98.7
Later re-adenoidectomy		
Yes	29	1.4
No	2076	98.6
	**Mean ± SD**	**Median**,** range**
Interval to re-adenoidectomy, months	12.8 ± 5.4	12.1, 3.6–24.5
Follow-up, months	1.0 ± 3.9	0, 0–32

*One patient had primary and secondary bleeding; **some patients received more than one treatment type.

The mean follow-up was 1.0 ± 3.9 months. 29 children (1.4%) needed later a re-adenoidectomy. The mean interval to re-adenoidectomy was 12.8 ± 5.4 months.

### Univariate and multivariate analysis of factors associated to complications and later re-adenoidectomy

The univariate analysis of associations between patients’ and treatment characteristics and postsurgical complications presented in Supplementary Tables 1 and Supplementary Table 2. When considering all surgery-specific postoperative complication together, male patients (*p* = 0.023), patients with Brodsky grade III/IV (*p* < 0.001), additional tonsillectomy (*p* < 0.001) and patients receiving antibiotics (*p* < 0.001) showed more frequently complications. Antibiotics were not more frequently used in children with comorbidity (CCI ≥ 1) than compared to the children without comorbidity (CCI = 0; *p* = 0.793). Use of perioperative antibiotics was the only factor associated to postoperative bleeding within 24 h after surgery (*p* < 0.001). In contrast, male gender (*p* = 0.039), underweight (*p* = 0.013), Brodsky grade III/IV (*p* = 0.009), additional tonsillectomy (*p* < 0.001) and patients receiving antibiotics (*p* < 0.001) had more frequently a bleeding later than 24 h after surgery. Comorbid patients (*p* = 0.042), Brodsky grade III/IV (*p* = 0.023), additional tonsillectomy (*p* < 0.001), and use of perioperative antibiotics (*p* < 0.001) showed more frequently a wound infection. Complications due the Clavien-Dindo classification were seen more frequently in comorbid patients (*p* = 0.021), children with underweight (*p* = 0.031), Brodsky grade III/IV (*p* < 0.001), additional tonsillectomy (*p* < 0.001), and when antibiotics were used (*p* < 0.001). Finally, later re-adenoidectomy was more frequently seen in children receiving an adenoidectomy with tonsil surgery (*p* = 0.020).

Multivariate binary logistic regressions were performed to estimate independent associations between patients’ and treatment characteristics versus the occurrence of complications (Tables 4 and 5) and versus the occurrence of later re-adenoidectomy (Table 6). Independent factors for any complication were comorbidity due to CCI (OR = 3.088; CI = 1.172–8.137; *p* = 0.023) and additional tonsillectomy when compared to adenoidectomy alone (OR = 6.949; CI = 2.069–23.242; *p* = 0.002).). In addition, patients receiving perioperative antibiotics had a higher and independent risk to develop complications (OR = 20.095; CI = 10.920-36.977; *p* < 0.001). The only independent factor related to bleeding after 24 h was additional tonsillectomy (OR = 52.141; CI = 7.772-349.818; *p* < 0.0001). There was no independent factor related to increased occurrence of a wound infection. The occurrence to complications classified by the CDC was independently related to occurrence of comorbidity defined by CCI (OR = 4.175; CI = 1.222–14.271; *p* = 0.023), underweight defined by the BMI (OR = 2.430; CI = 1.198–6.571; *p* = 0.040), highly associated to additional tonsillectomy (OR = 11.177; CI = 2.098–59.548; *p* < 0.0001). Patients receiving perioperative antibiotics had also a higher independent risk of CDC complications (OR = 13.251; CI = 5.695–30.834; *p* < 0.001).

**Table 4:** Multivariate binary logistic regression analysis of associations between patients’ and treatment characteristics and postsurgical complications.

Parameter	OR	95% CI	*p*
**Model 1: Any complication (yes/no)**			
Charlson Comorbidity Index			
0	1	Reference	
1+	3.088	1.172–8.137	**0.023**
Brodsky grade			
No obstruction, grade 0	1	Reference	
≤ 25% obstruction, grade I	0.563	0.063-5.000	0.606
26–50% obstruction, grade II	0.397	0.142–1.111	0.078
51–75% obstruction, grade III	0.404	0.135–1.209	0.105
> 75% obstruction, grade IV	0.312	0.082–1.181	0.086
Surgery types			
Adenoidectomy, solitary	1	Reference	
Adenoidectomy + tonsillotomy	1.638	0.736–3.643	0.227
Adenoidectomy + tonsillectomy	6.949	2.069–23.242	**0.002**
Perioperative antibiotics			
No	1	Reference	
Yes	20.095	10.920-36.977	**< 0.001**
**Model 2: Bleeding after 24 h (yes/no)**			
Gender			
Female	1	Reference	
Male	2.935	0.816–10.562	0.099
BMI percentiles			
3.0 (underweight)	1	Reference	
10.0	NA		
25.0	2.697	0.452–16.080	0.276
50.0	1.694	0.389–7.387	0.483
75.0	NA		
90.0 (overweight)	1.722	0.338–8.772	0.513
97.0 (obesity)	0.687	0.130–3.626	0.658
99.5 (extreme obesity)	3.514	0.366–33.710	0.276
Brodsky grade			
No obstruction, grade 0	1	Reference	
≤ 25% obstruction, grade I	0.301	0.014–6.503	0.444
26–50% obstruction, grade II	0.741	0.068–8.042	0.806
51–75% obstruction, grade III	0.572	0.048–6.777	0.658
> 75% obstruction, grade IV	0.178	0.012–2.612	0.208
Surgery type			
Adenoidectomy, solitary	1	Reference	
Adenoidectomy + tonsillotomy	2.993	0.570-15.722	0.195
Adenoidectomy + tonsillectomy	52.141	7.772-349.818	**< 0.001**
Perioperative antibiotics			
No	1	Reference	
Yes	2.041	0.468–8.895	0.342

NA = not applicable.

**Table 5:** Multivariate binary logistic regression analysis of associations between patients’ and treatment characteristics and postsurgical complications.

Parameter	OR	95% CI	*p*
**Model 3: Wound infection (yes/no)**			
Charlson Comorbidity Index			
0	1	Reference	
1+	NA		
BMI percentiles			
3.0 (underweight)	1	Reference	
10.0	NA		
25.0	NA		
50.0	NA		
75.0	NA		
90.0 (overweight)	NA		
97.0 (obesity)	NA		
99.5 (extreme obesity)	NA		
Brodsky grade			
No obstruction, grade 0	1	Reference	
≤ 25% obstruction, grade I	NA		
26–50% obstruction, grade II	NA		
51–75% obstruction, grade III	3.000	0.084-107.447	0.547
> 75% obstruction, grade IV	NA		
Surgery type			
Adenoidectomy, solitary	NA		
Adenoidectomy + tonsillotomy	NA		
Adenoidectomy + tonsillectomy	NA		
Perioperative antibiotics			
No	1	Reference	
Yes	NA		
**Model 4: CDC complications (grade 0/grade I+)**			
Charlson Comorbidity Index			
0	1	Reference	
1+	4.175	1.222–14.271	**0.023**
BMI percentiles, underweight			
No	1	Reference	
Yes	2.430	1.198–6.571	**0.040**
Brodsky grade			
No obstruction, grade 0	1	Reference	
≤ 25% obstruction, grade I	0.555	0.060–5.111	0.603
26–50% obstruction, grade II	3.818	0.662–22.014	0.134
51–75% obstruction, grade III	1.112	0.252–4.911	0.888
> 75% obstruction, grade IV	0.756	0.132–4.312	0.752
Surgery type			
Adenoidectomy, solitary	1	Reference	
Adenoidectomy + tonsillotomy	2.923	0.797–10.727	0.106
Adenoidectomy + tonsillectomy	11.177	2.098–59.548	**0.005**
Perioperative antibiotics			
No	1	Reference	
Yes	13.251	5.695–30.834	**< 0.001**

**Table 6:** Multivariate binary logistic regression analysis of associations between patients’ and treatment characteristics and risk of re-adenoidectomy.

Parameter	OR	95% CI	*p*
**Model 5: Re-adenoidectomy (yes/no)**			
Brodsky grade			
No obstruction, grade 0	1	Reference	
≤ 25% obstruction, grade I	7.105	1.406–35.901	**0.018**
26–50% obstruction, grade II	4.544	1.297-15.9123	**0.018**
51–75% obstruction, grade III	2.451	0.531–11.302	0.250
> 75% obstruction, grade IV	11.742	1.406–98.028	**0.023**
Surgery type			
Adenoidectomy, solitary	1	Reference	
Adenoidectomy + tonsillotomy	0.105	0.017–0.646	**0.015**
Adenoidectomy + tonsillectomy	2.78899E-08	NA	0.998

OR = Odds ratio; CI = confidence interval; significant p-values (*p* < 0.05) in bold.

The probability of later need of re-adenoidectomy was independently associated to the grade of oropharyngeal obstruction defined by the Brodsky grade (≤ 25% obstruction, grade I: OR = 7.105; CI = 1.406–35.901; *p* = 0.018; 26–50% obstruction, grade II: OR = 4.544; CI = 1.297-15.9123; *p* = 0.018; 51–75% obstruction, grade III; OR = 2.451; CI = 0.531–11.302; *p* = 0.250; >75% obstruction, grade IV: OR = 11.742; CI = 1.406–98.028; *p* = 0.023). Hence, the highest probability for later re-adenoidectomy was seen in children with Brodsky grade II. And the re-adenoidectomy rate was lower in case of prior adenoidectomy in combination with tonsillotomy compared to adenoidectomy alone (OR = 0.105; CI = 0.017–0.646: *p* = 0.015).

### Incidence rates of surgery and of complications

**Fig. 1:**
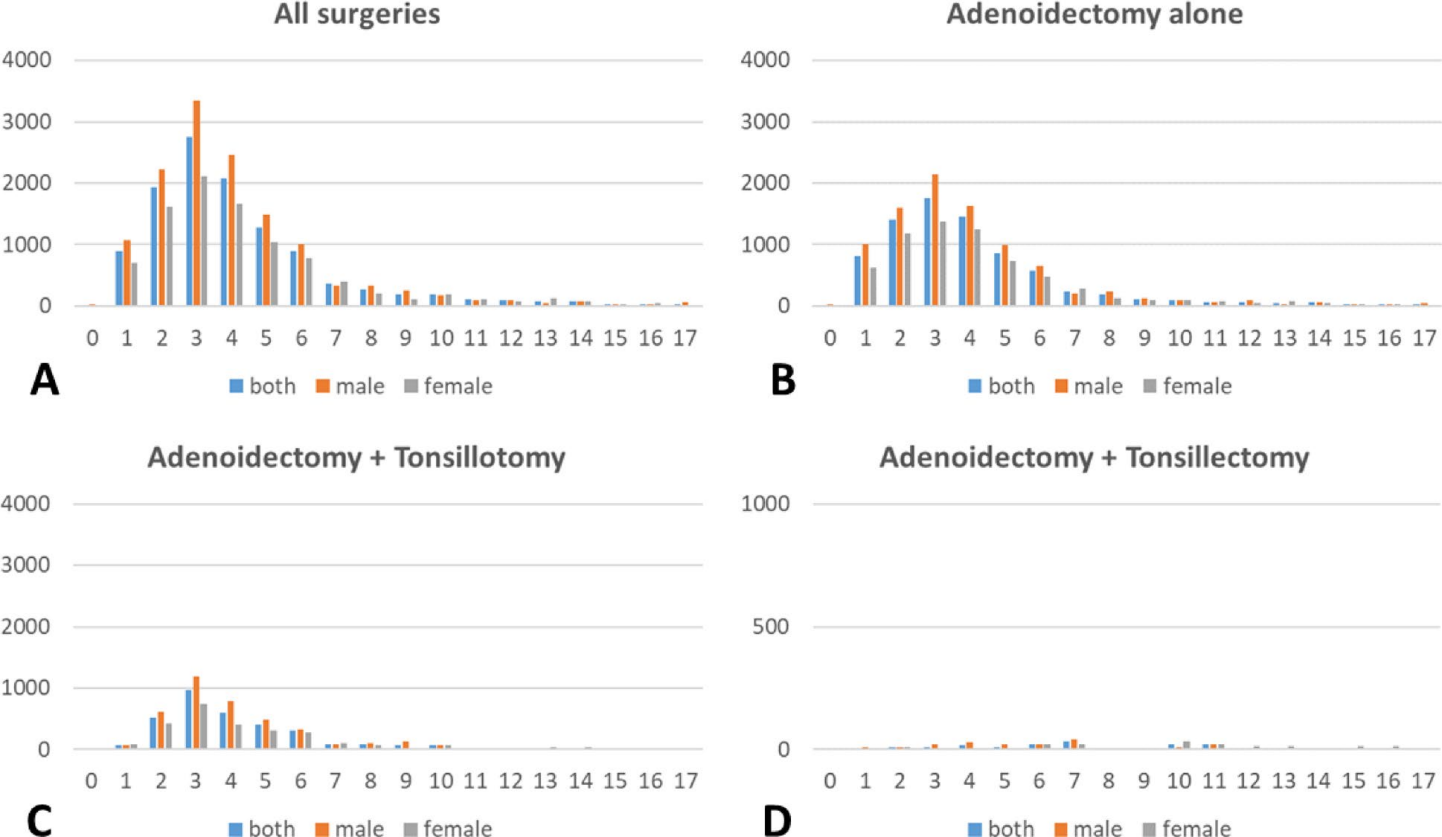
Incidence of adenoidectomy (surgical rate) per 100,000 Thuringian children in 2019 per year of age for all children (blue), and separately for male (orange) and female (grey) children. **A**: for all surgeries. **B**: for adenoidectomy alone. **C**: Combined surgery of adenoidectomy and tonsillotomy. **D**: Combined surgery of adenoidectomy and tonsillectomy. X-axis: Age in years. Y-axis: absolute number per 100,000.

**Fig. 2:**
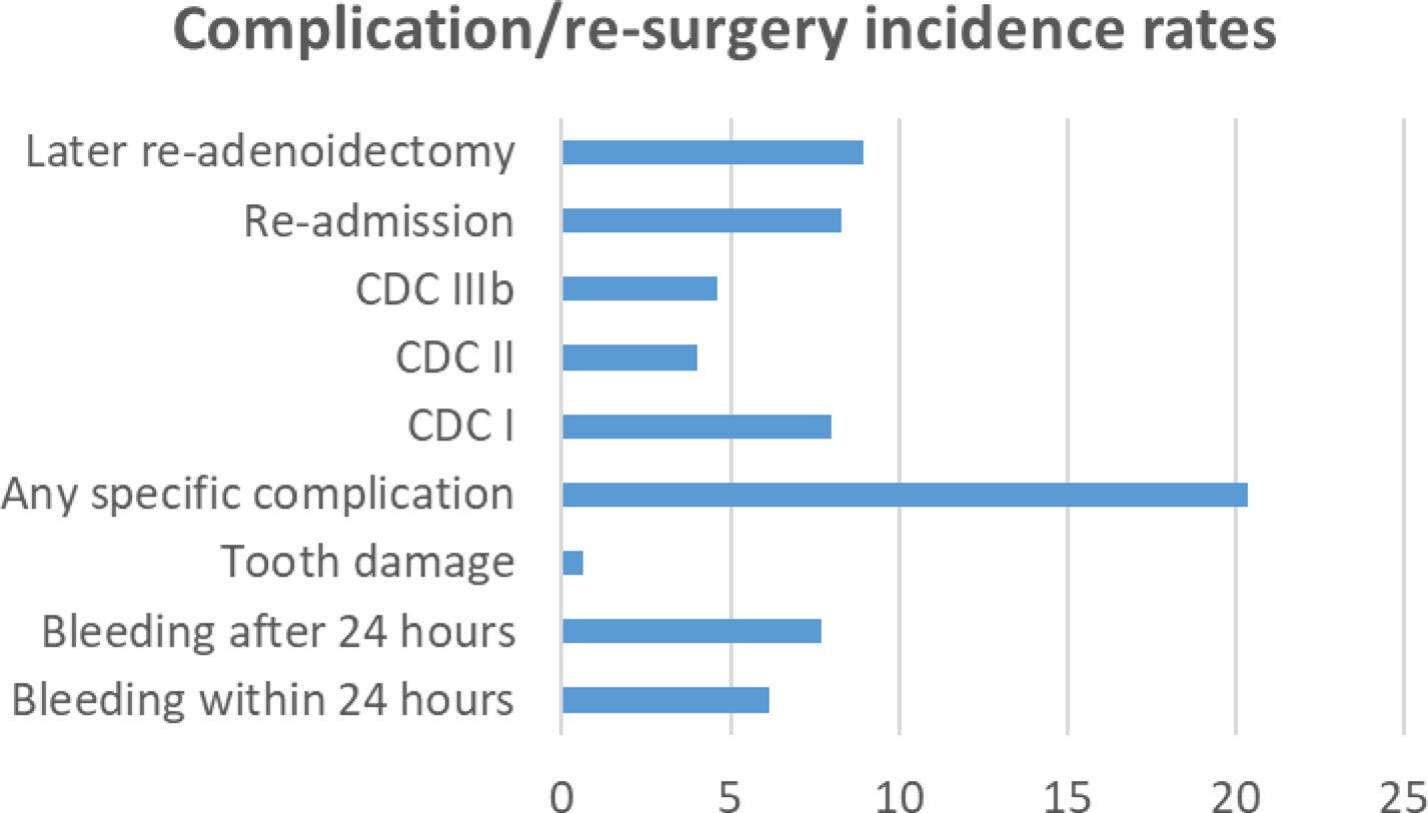
Incidence of perioperative complications and need for later re-adenoidectomy per 100,000 Thuringian population in 2019. Re-admission means re-admission because of complications. X-axis: absolute number per 100,000 children. Y-axis: the most important types of complication. CDC = Clavien-Dindo classification.

Thuringia had 324,392 (male: 166,707; female: 157,689) underage habitants in 2019. The highest adenoidectomy rates was reached for both genders at the age of 3 years with 3344.3 and 2116.5, respectively, per 100,000 children (**Supplementary Table 3)**. Up to the age of 6 years, the surgery rates were always higher in male compared to female children (Fig. 1). The rates were much lower for combined surgery (plus tonsillotomy or tonsillectomy), but peaked also at the age of 3 years and the male predominance up to the age of six years was also seen for the combined surgeries. The overall complication rate was 20.3/100,000 population (Fig. 2). The rate for bleeding within 24 h was 6.2/100,000, after 24 h 7.7/100,000, and for tooth damage 0.6/100,000. Classified by CDC, CDC grade I, II, and IIIb, respectively, occurred in 8.0, 4.0, and 4.6 per 100,000 population. 8.9/100,000 children needed a later re-adenoidectomy.

## Discussion

This population-based analysis on 2105 adenoidectomies from one year revealed that complications after surgery of this very frequent surgery are very low. Hence, adenoidectomy is very safe surgery. The most important complication, i.e. postoperative bleeding needing re-surgery, occurred in 1.1% of all cases and in 0.7% of the cases of solitary adenoidectomy without palatine tonsil surgery. The overall complication rate was 20.3/100,000 population. Additional tonsillectomy was associated to higher risk of postoperative bleeding. Comorbidity was associated to higher likelihood of healing wound infection. CDC complications were more frequently seen in comorbid patients, underweight children, and again when additional tonsillectomy was performed. Practical consequences of these results could be should be a reminder to parents that surgery on the palatine tonsils is the more risky procedure, closer monitoring of all risk groups, but also a pre-operative nutritional screening and counselling of parents with underweight children.

In accordance to the large Swedish register study on 40,829 children, the highest age related incidence is seen was 2–4 years for both genders. Boys had higher incidence rates than girls at least up to the age of 6 years^15^. The age peak for surgery might be explained by dynamic immunological growth of the adenoids seen between ages 3 and 6 years, which is be related to relatively slower growth of the nasopharyngeal cavity^16^. In contrast, it is unclear why male patients undergo surgery more often. This gender difference have been reported also by others, without being able to explain this gender dimorphism^15,17,18^. It seems to be that multiple factors contribute to this gender difference^19^. Relevant craniofacial and pharyngeal differences seem to be unlikely^20^. The social perception of symptoms like snoring might be different for boys and girls. This has not yet been explored in detail^19^.

In our previous study from Thuringia, the peak incidence was at 3 years with a rate of 2792/100,000 children in 2009. Now for 2019 it was 2747/100,000 children, hence the rate had not changed. In Sweden the rate was also stable between 2004 and 2013 ^5^. As population-based studies are rare, not much is known about the worldwide variability. The numbers are varying from 170/100,000 in Canada, 440/100,000 in Norway, to 687/100,000 in the United States, 290/100,000 in Belgium and 1330/100,000 in Finland up to 1460/100,000 in Sweden and now 2747/100,000 in Germany^15,18,21^. We can only speculate as to why this is the case. In general, there are large international differences in adenotonsillectomy rates^22^. A well-organized and socially equitable access to the healthcare system can certainly only explain a small part of this^23^. It is possible that the criteria for the indication of adenoidectomy are less strictly defined in Germany than in other countries.

The largest population-based analysis of complications also comes from the Swedish National Register. In 51,746 children who underwent adenoidectomy from 2007 to 2017, the rate of outpatient visits due to postoperative hemorrhage (96% outpatient surgery) was 0.1%, and the re-admissions due to postoperative hemorrhage was also 0.1% ^6^. It is difficult to compare the Swedish results directly with the present study because hemorrhages occurring during hospital stay and before discharge were not reported to the Swedish register. This might explain why the present rate of 0.7% appeared to be also low but higher than in Sweden. For 2009, we reported a rate of 1% for all bleedings^17^i.e. the rate remained unchanged and is in the range (0.2–0.8%) of other studies^3,24–26^. In general, and also due to a meta-analysis from 2015, the most frequent early complication after adeno-tonsillectomy is postoperative hemorrhage. The cause is almost always the tonsil surgery and not the adenoid surgery^27^. As adenoid and tonsil surgery are frequently combined as adenotonsillectomy, one has to be careful when interpreting complication data, especially when the relation to the prior tonsil and adenoid surgery is not considered separately^4^. And comorbidity - as in the present study- is in general a well-known risk factor for postoperative complications after pediatric adeno-tonsillectomy^28^. Overall, the complication rate according to adenoidectomy is certainly low. This could lead to this being trivialized when informing the parents. Parents of children with comorbidity should be made aware of the increased risk of complications in children with comorbidity. And the treating physicians should pay particular attention to children who receive perioperative antibiotics (the comorbidity may also have led to this) in order to detect complications as early as possible.

The re-adenoidectomy rate was 1.4%. This resulted in a population rate of 8.9 re-adenoidectomies per100,000 children. This rate is lower than for Taiwan between 2000 and 2007. Out of 10,396 Taiwanese children who underwent primary adenoidectomy, 2.6% received a re-adenoidectomy^6^. Out of 41,401 children with primary adenoidectomy from the Swedish National registry10.7% underwent a second surgery, but they did not differentiate between second surgery at the adenoids from surgery at the tonsils^29^. But the calculated incidence rate are presented separately, given with 7.2, 2.5, and 1.4 per 100,000 children for re-adenoidectomy, re-adenotonsillotomy, and re-adenotonsillectomy, respectively. Hence, this is in the same range as in the present study. The risk for re-adenoidectomy was also in Sweden higher for children with solitary adenoidectomy as primary surgery.

The present study has some limitations. Due to the retrospective design, decision making could not be analyzed, i.e. we could not analyze why, for instance, adenoidectomy was combined with tonsil surgery or not. Furthermore, only associations between adenoidectomy and complication could be analyzed but no causalities. For instance, the use of antibiotics was related to higher probability of complications. However, the use of antibiotics was most likely not the cause of complications, but their consequence.

In conclusion, this population-based analysis demonstrated pediatric adenoidectomy is a very safe surgery. The major factor for complications, mainly it is postoperative bleeding, is not the adenoidectomy itself but if it is combined with tonsil surgery. From the epidemiological point of view, studies are needed to clarify why adenoidectomy is more frequent in male patients. Furthermore, we need a better understanding of the variability of adenoidectomy rates worldwide. At best, prospective multicenter studies are needed to study approaches to reduce complications among high-risk patient populations, for instance, to analyze if closer monitoring of children at risk or if nutritional support for underweight children could further reduce complications of pediatric adenoidectomy.

## Supplementary Information

Below is the link to the electronic supplementary material.

Supplementary Material 1

## Data Availability

The datasets used and analyzed during the current study are available from the corresponding author on reasonable request.
