# Supplementary material for: Pediatric adenoidectomy is safe surgery with a low complication rate: a population-based study
Source: Sci Rep. 2025 Jul 31;15:27967. doi: 10.1038/s41598-025-13803-9 (PMC12314081; doi:10.1038/s41598-025-13803-9)
Supplement: Supplementary file 1 — Supplementary Material 1 [file 41598_2025_13803_MOESM1_ESM.docx]

**Supplementary material**

**Pediatric adenoidectomy is safe surgery with a low complication rate: a population-based study**

Hannah Losgar, Daniel Boeger, Jens Buentzel, Kerstin Hoffmann, Jiri Podzimek, Holger Kaftan, Andreas Mueller, Sylvia Tresselt, Katharina Geißler, Orlando Guntinas-Lichius

**3 Supplementary Tables**

**Supplementary Tables**

**Supplementary Table 1**

| **Supplementary Table 1.** Univariate analysis of association of patients’ and treatment characteristics on postsurgical complications I. | | | | | | | | | | |
| --- | --- | --- | --- | --- | --- | --- | --- | --- | --- | --- |
|  | **Any postsurgical complication** | | | **Bleeding within 24 hours** | | | **Bleeding after 24 hours** | | |  |
| **Parameter** | **Yes** | **No** | **p** | **Yes** | **No** | **p** | **Yes** | **No** | **p** |  |
| Gender |  |  | 0.569 |  |  | 0.364 |  |  | **0.039** |  |
| Male | 43 | 1218 |  | 10 | 1251 |  | 20 | 1241 |  |  |
| Female | 24 | 819 |  | 10 | 834 |  | 5 | 839 |  |  |
| Charlson Comorbidity Index |  |  | **0.023** |  |  | 0.248 |  |  | 0.416 |  |
| 0 | 7 | 90 |  | 18 | 1990 |  | 2 | 95 |  |  |
| 1+ | 61 | 1947 |  | 2 | 95 |  | 23 | 1985 |  |  |
| BMI percentiles |  |  | 0.336 |  |  | 0.123 |  |  | **0.020** |  |
| 3.0 (underweight) | 7 | 120 |  | 3 | 124 |  | 5 | 122 |  |  |
| 10.0 | 3 | 149 |  | 3 | 149 |  | 0 | 152 |  |  |
| 25.0 | 3 | 232 |  | 1 | 234 |  | 2 | 233 |  |  |
| 50.0 | 9 | 263 |  | 3 | 269 |  | 4 | 268 |  |  |
| 75.0 | 6 | 259 |  | 0 | 265 |  | 1 | 264 |  |  |
| 90.0 (overweight) | 8 | 179 |  | 4 | 183 |  | 4 | 183 |  |  |
| 97.0 (obesity) | 4 | 91 |  | 0 | 95 |  | 4 | 91 |  |  |
| 99.5 (extreme obesity) | 5 | 121 |  | 2 | 124 |  | 1 | 125 |  |  |
| BMI percentiles, underweight |  |  | 0.098 |  |  | 0.152 |  |  | **0.013** |  |
| Yes | 7 | 120 |  | 3 | 124 |  | 5 | 122 |  |  |
| No | 38 | 1294 |  | 13 | 1319 |  | 10 | 1316 |  |  |
| BMI percentiles, overweight+ |  |  | 0.136 |  |  | 0.393 |  |  | 0.126 |  |
| Yes | 17 | 391 |  | 6 | 402 |  | 9 | 399 |  |  |
| No | 28 | 1023 |  | 10 | 1041 |  | 12 | 1039 |  |  |
| Setting |  |  | 0.087 |  |  | 0.236 |  |  | 0.609 |  |
| Inpatient | 67 | 1901 |  | 20 | 1948 |  | 24 | 1944 |  |  |
| Outpatient | 1 | 136 |  | 0 | 137 |  | 1 | 136 |  |  |
| Brodsky grade |  |  | **0.003** |  |  | 0.670 |  |  | **0.009** |  |
| No obstruction, grade 0 | 6 | 491 |  | 2 | 495 |  | 1 | 496 |  |  |
| ≤25% obstruction, grade I | 1 | 72 |  | 0 | 73 |  | 1 | 72 |  |  |
| 26-50% obstruction, grade II | 14 | 573 |  | 6 | 581 |  | 5 | 582 |  |  |
| 51-75% obstruction, grade III | 28 | 598 |  | 6 | 620 |  | 8 | 618 |  |  |
| >75% obstruction, grade IV | 10 | 163 |  | 2 | 171 |  | 6 | 167 |  |  |
| Surgery type |  |  | **<0.001** |  |  | 0.855 |  |  | **<0.001** |  |
| Adenoidectomy, solitary | 30 | 1429 |  | 14 | 1445 |  | 7 | 1452 |  |  |
| Adenoidectomy + tonsillotomy | 31 | 583 |  | 6 | 608 |  | 12 | 602 |  |  |
| Adenoidectomy + tonsillectomy | 7 | 25 |  | 0 | 32 |  | 6 | 26 |  |  |
| Perioperative antibiotics |  |  | **<0.001** |  |  | **<0.001** |  |  | **<0.001** |  |
| Yes | 27 | 70 |  | 7 | 90 |  | 5 | 92 |  |  |
| No | 41 | 1967 |  | 13 | 1995 |  | 20 | 1988 |  |  |
|  | **M±SD** | **M±SD** | **p** | **M±SD** | **M±SD** | **p** | **M±SD** | **M±SD** | **p** |  |
| Age, years | 4.3±0.3 | 4.2±0.1 | 0.581 | 4.6±2.8 | 4.2±2.6 | 0.616 | 4.5±0.6 | 4.2±0.1 | 0.507 |  |
| BMI | 16.3±0.5 | 16.3±0.1 | 0.493 | 15.7±3.4 | 16.3±3.9 | 0.563 | 16.0±0.8 | 16.3±3.9 | 0.728 |  |

*±adenoidectomy; M = mean; SD = standard deviation;; significant p-values (p<0.05) in bold.

**Supplementary Table 2**

| **Supplementary Table 2.** Univariate analysis of association of patients’ and treatment characteristics on postsurgical complications II. | | | | | | | | | | |
| --- | --- | --- | --- | --- | --- | --- | --- | --- | --- | --- |
|  | **Wound infection** | | | **CDC classification** | | | **Re-adenoidectomy** | | |  |
| **Parameter** | **Yes** | **No** | **p** | **≥1** | **0** | **p** | **Yes** | **No** | **p** |  |
| Gender |  |  | 0.938 |  |  | 0.922 |  |  | 0.535 |  |
| Male | 13 | 1248 |  | 32 | 1229 |  | 19 | 1242 |  |  |
| Female | 9 | 835 |  | 22 | 833 |  | 10 | 834 |  |  |
| Charlson Comorbidity Index |  |  | **0.042** |  |  | **0.021** |  |  | 0.233 |  |
| 0 | 3 | 94 |  | 6 | 91 |  | 0 | 97 |  |  |
| 1+ | 19 | 1989 |  | 49 | 1960 |  | 29 | 1979 |  |  |
| BMI percentiles |  |  | **0.027** |  |  | 0.154 |  |  | 0.848 |  |
| 3.0 (underweight) | 0 | 127 |  | 7 | 120 |  | 2 | 125 |  |  |
| 10.0 | 0 | 152 |  | 3 | 149 |  | 2 | 150 |  |  |
| 25.0 | 0 | 235 |  | 2 | 233 |  | 3 | 232 |  |  |
| 50.0 | 1 | 271 |  | 7 | 265 |  | 5 | 267 |  |  |
| 75.0 | 5 | 260 |  | 4 | 261 |  | 1 | 264 |  |  |
| 90.0 (overweight) | 0 | 187 |  | 7 | 180 |  | 2 | 185 |  |  |
| 97.0 (obesity) | 0 | 95 |  | 4 | 91 |  | 2 | 93 |  |  |
| 99.5 (extreme obesity) | 2 | 124 |  | 4 | 122 |  | 1 | 125 |  |  |
| BMI percentiles, underweight |  |  | 0.381 |  |  | **<0.031** |  |  | 0.716 |  |
| Yes | 0 | 127 |  | 7 | 120 |  | 2 | 125 |  |  |
| No | 8 | 1324 |  | 31 | 1301 |  | 16 | 1316 |  |  |
| BMI percentiles, overweight+ |  |  | 0.851 |  |  | 0.109 |  |  | 0.986 |  |
| Yes | 2 | 406 |  | 15 | 393 |  | 13 | 1038 |  |  |
| No | 6 | 1045 |  | 23 | 1028 |  | 5 | 403 |  |  |
| Setting |  |  | 0.213 |  |  | 0.160 |  |  | 0.501 |  |
| Inpatient | 22 | 1946 |  | 53 | 1915 |  | 28 | 1940 |  |  |
| Outpatient | 0 | 137 |  | 2 | 136 |  | 1 | 136 |  |  |
| Brodsky grade |  |  | **0.023** |  |  | **<0.001** |  |  | **0.009** |  |
| No obstruction, grade 0 | 2 | 495 |  | 5 | 492 |  | 3 | 494 |  |  |
| ≤25% obstruction, grade I | 0 | 73 |  | 1 | 72 |  | 3 | 70 |  |  |
| 26-50% obstruction, grade II | 3 | 584 |  | 7 | 580 |  | 14 | 573 |  |  |
| 51-75% obstruction, grade III | 13 | 613 |  | 24 | 602 |  | 4 | 622 |  |  |
| >75% obstruction, grade IV | 3 | 170 |  | 10 | 163 |  | 2 | 171 |  |  |
| Surgery type |  |  | **<0.001** |  |  | **<0.001** |  |  | **0.020** |  |
| Adenoidectomy, solitary | 7 | 1452 |  | 19 | 1440 |  | 27 | 1432 |  |  |
| Adenoidectomy + tonsillotomy | 14 | 600 |  | 30 | 584 |  | 2 | 612 |  |  |
| Adenoidectomy + tonsillectomy | 1 | 31 |  | 5 | 27 |  | 0 | 32 |  |  |
| Perioperative antibiotics |  |  | **<0.001** |  |  | **<0.001** |  |  | 0.233 |  |
| Yes | 16 | 81 |  | 20 | 77 |  | 0 | 97 |  |  |
| No | 6 | 2002 |  | 34 | 1974 |  | 29 | 1979 |  |  |
|  | **M±SD** | **M±SD** | **p** | **M±SD** | **M±SD** | **p** | **M±SD** | **M±SD** | **p** |  |
| Age, years | 3.6±2.0 | 4.2±0.1 | 0.331 | 4.2±2.8 | 4.2±2-6 | 0.685 | 3.0±0.4 | 4.2±0.1 | 0.194 |  |
| BMI | 18.0±1.5 | 16.3±0.1 | 0.496 | 16.2±3.8 | 16.3±3.9 | 0.677 | 15.6±0.4 | 16.3±3.9 | 0.192 |  |

**Supplementary Table 3**

| **Supplementary Table 3.** Surgical rates (Incidence rates) per 100,000 children. | | | | | | | | | | | | |
| --- | --- | --- | --- | --- | --- | --- | --- | --- | --- | --- | --- | --- |
|  | **All surgeries** | | | **Adenoidectomy alone** | | | **Adenoidectomy + tonsillotomy** | | | **Adenoidectomy + tonsillectomy** | | |
| **Age (years)** | **All** | **Male** | **Female** | **All** | **Male** | **Female** | **All** | **Male** | **Female** | **All** | **Male** | **Female** |
| 0 | 17.6 | 23.1 | 12.0 | 17.6 | 23.1 | 12.0 | 0.0 | 0.0 | 0.0 | 0.0 | 0.0 | 0.0 |
| 1 | 892.6 | 1076.7 | 698.7 | 814.5 | 1000.5 | 618.6 | 72.5 | 65.3 | 80.2 | 72.5 | 65.3 | 80.2 |
| 2 | 1927.5 | 2223.2 | 1618.6 | 1399.9 | 1601.5 | 1189.2 | 516.9 | 611.1 | 418.4 | 516.9 | 611.1 | 418.4 |
| 3 | 2747.4 | 3344.3 | 2116.5 | 1766.2 | 2136.7 | 1374.6 | 970.6 | 1187.0 | 741.9 | 970.6 | 1187.0 | 741.9 |
| 4 | 2073.4 | 2466.0 | 1655.6 | 1450.9 | 1637.2 | 1252.6 | 606.7 | 798.1 | 403.0 | 606.7 | 798.1 | 403.0 |
| 5 | 1274.7 | 1492.7 | 1044.3 | 862.1 | 988.3 | 728.8 | 402.0 | 483.8 | 315.5 | 402.0 | 483.8 | 315.5 |
| 6 | 898.9 | 1010.1 | 781.4 | 572.5 | 656.0 | 484.3 | 305.0 | 333.2 | 275.1 | 305.0 | 333.2 | 275.1 |
| 7 | 368.1 | 336.6 | 401.5 | 243.6 | 210.4 | 278.8 | 92.0 | 84.2 | 100.4 | 92.0 | 84.2 | 100.4 |
| 8 | 270.2 | 337.8 | 199.4 | 183.8 | 232.2 | 132.9 | 86.5 | 105.6 | 66.5 | 86.5 | 105.6 | 66.5 |
| 9 | 185.8 | 257.8 | 111.2 | 109.3 | 128.9 | 89.0 | 76.5 | 128.9 | 22.2 | 76.5 | 128.9 | 22.2 |
| 10 | 182.0 | 173.1 | 191.4 | 93.8 | 97.3 | 90.1 | 66.2 | 64.9 | 67.6 | 66.2 | 64.9 | 67.6 |
| 11 | 104.7 | 96.0 | 114.1 | 66.2 | 53.3 | 79.9 | 16.5 | 21.3 | 11.4 | 16.5 | 21.3 | 11.4 |
| 12 | 86.1 | 88.3 | 83.7 | 68.8 | 88.3 | 47.8 | 11.5 | 0.0 | 23.9 | 11.5 | 0.0 | 23.9 |
| 13 | 82.3 | 45.4 | 121.7 | 52.9 | 34.1 | 73.0 | 23.5 | 11.4 | 36.5 | 23.5 | 11.4 | 36.5 |
| 14 | 80.5 | 78.1 | 82.9 | 57.5 | 66.9 | 47.4 | 23.0 | 11.2 | 35.5 | 23.0 | 11.2 | 35.5 |
| 15 | 34.4 | 33.5 | 35.4 | 28.7 | 33.5 | 23.6 | 0.0 | 0.0 | 0.0 | 0.0 | 0.0 | 0.0 |
| 16 | 34.8 | 22.5 | 47.9 | 23.2 | 22.5 | 23.9 | 5.8 | 0.0 | 12.0 | 5.8 | 0.0 | 12.0 |
| 17 | 28.7 | 55.4 | 0.0 | 23.0 | 44.3 | 0.0 | 5.7 | 11.1 | 0.0 | 5.7 | 11.1 | 0.0 |
| All | 648.9 | 756.4 | 535.2 | 449.8 | 519.5 | 376.1 | 189.3 | 226.1 | 150.3 | 189.3 | 226.1 | 150.3 |
